# Wuzi Yanzong pill, a Chinese polyherbal formula, alleviates testicular damage in mice induced by ionizing radiation

**DOI:** 10.1186/s12906-016-1481-6

**Published:** 2016-12-07

**Authors:** Hai-Jie Ji, Dong-Mei Wang, Yu-Peng Wu, Yan-Yan Niu, Li-Li Jia, Bi-Wang Liu, Qian-Jin Feng, Ma-Li Feng

**Affiliations:** 1 Shanxi Province Academy of Traditional Chinese Medicine, Taiyuan, 030012 China; 2 State Key Laboratory of Bioactive Substance and Function of Natural Medicines, Institute of Materia Medica, Chinese Academy of Medical Sciences and Peking Union Medical College, Beijing, 100050 China; 3 Shanxi University of Traditional Chinese Medicine, Taiyuan, 030024 China

**Keywords:** Wuzi Yanzong pill, Ionizing irradiation, Testicular damage

## Abstract

**Background:**

Chinese medicine Wuzi Yanzong pill (WZYZP) was firstly documented in ancient Chinese medical works “She Sheng Zhong Miao Fang” by Shi-Che Zhang in 1550 AD. The traditional herbal formula is widely used in treating nephrasthenia lumbago, prospermia, erectile dysfunction and male sterility. The present study was to explore the effects of WZYZP on ionizing irradiation-induced testicular damage in mice.

**Methods:**

The pelvic region of male mice was exposed to X-rays for inducing testicular damage. The effects of WZYZP on testicular damage were evaluated in terms of testes weight, sperm quantity and motility, testes oxidative status and serum hormone levels. The alterations in testicular structure were examined by hematoxylin-eosin staining. Additionally, changes in proliferating cell nuclear antigen (PCNA) expression of testes were explored by western blot.

**Results:**

Pelvic exposure to x-ray induced reduction in testes weight and sperm quality, along with oxidative stress and abnormal testicular architecture in testes. Oral administration of WZYZP for 3 weeks markedly increased testes weight, sperm quantity and motility, and attenuated testicular architecture damage. Meanwhile, WZYZP treatment significantly reversed the reduction of serum testosterone, and decreased testes malondialdehyde (MDA) and Oxidative stress index (OSI) relative to the radiated mice. Additionally, WZYZP effectively prevented the downregulation of PCNA expression in testes induced by x-ray irradiation.

**Conclusion:**

These findings suggest WZYZP exhibits ameliorating effects against ionizing irradiation-induced testicular damage in mice, which may be related to its antioxidation.

## Background

It is estimated that infertility affects 15% of all couples trying to conceive and male infertility is implicated in almost half of these cases [1]. As one of the most radiosensitive organs, spermatogenesis can be significantly impaired by ionizing irradiation and the quantity of sperm is reduced in irradiated testes, causing temporary or even permanent infertility [2]. Previous clinical study has demonstrated that oligozoospermia, aspermia, infertility and sexual dysfunction often occur during ionizing irradiation treatment in male patients with rectal cancer [3]. Also, the reproductive value of male subjects working in high voltage electric stations, mobile communication base stations and other electromagnetic environments has significantly decreased [4]. So far, a variety of chemical and biological compounds have been screened as radio-protectors worldwide [5–7]. However, these molecular or synthetic drugs are limited due to their unacceptable level of the toxicity to one or more vital body systems at the effective concentration.

Chinese herbal prescriptions, the basic form of clinical application of traditional Chinese medicine for thousands of years, have been proven by clinical practice to play a positive role in human health. Wuzi Yanzong pill (WZYZP), a kidney-reinforcing Chinese herbal formula, was firstly documented in ancient Chinese medical works “She Sheng Zhong Miao Fang” by Shi-Che Zhang in 1550 AD. WZYZP consisting of five medicinal plants (Table 1) is widely used to treat syndrome of kidney deficiency and damage of essence, including impotence, sterility, spermatorrhea, premature ejaculation and lumbago. Clinical observation has showed that WZYZP could enhance the spermatic density and motility, and raise the pregnant rate in spouses [8]. Previously some basic study indicated that WZYZP improves sperm quality in tripterygium glycosides induced oligoasthenospermia rats [9] and promotes spermatogenesis by modulating the secretory function of Sertoli cells [10]. However, there is a scarcity of information concerning its protective effect against reproductive cells damage.

**Table 1:** Composition of WuziYanzong prescription (WZYZP)

Botanical name	Part used	Proportion (%)
*Lycium barbarum L.*	Fruit	35
*Cuscuta chinensis Lam.*	Fruit	35
*Rubus chingii Hu.*	Fruit	17
*Schizandra chinensis (Turcz.) Baill.*	Fruit	5
*Plantago asiatica L.*	Fruit	8

In this study, a widely accepted model of testis exposure to x-ray in male mouse was used to evaluate the possible therapeutic potential of WZYZP on testicular damage, and the underlying mechanisms were also explored.

## Methods

### Animals

Adult male Kunming mice aged 6 weeks were housed in groups of six per cage at a temperature of 22 ± 1 °C with a 12 h light–dark cycle (light on 7 a.m.–7 p.m.), and had free access to the food and water for 7 days prior to irradiation. All the experimental and animal handling procedures were approved by the Faculty Committee on the Use of Live Animals in Teaching and Research in the Shanxi province academy of traditional Chinese medicine (Taiyuan, China).

### Drugs

WuziYanzong prescription is composed of five different Chinese medicinal herbs (Table 1). In this study, all traditional Chinese herbs were purchased from Tong Ren Tang Chinese Medicine Co., Ltd. (Beijing, China) and ground to a fine powder. WZYZP powder was dissolved in 0.5% CMC and administrated p.o. at a volume of 20 ml/kg.

### Testicular x-ray irradiation

Testicular irradiation was performed according to the previous report [11]. Initially, mice were anesthetized with chloral hydrate (350 mg/kg, ip) and immobilized in the supine position. Only the testes region was irradiated with a single dose of 4 Gy x-rays while the body was shielded with 1-mm thick lead shields. Irradiation was carried out with a Gilardoni x-ray machine (15 mA, 250 kV; dose rate 0.96 Gy/min). Mice in control group were underwent the same anesthetic procedure but no irradiation. After irradiation all animals were returned to the animal facility.

### Experimental design

The irradiated mice were randomly divided into 3 groups: (1) irradiated mice treated with distilled water; (2) irradiated mice treated with WZYZP 0.25 g/kg; (3) irradiated mice treated with WZYZP 1.0 g/kg. The control mice treated with distilled water were served as control group. Each group consisted of 12 mice with identical mean body weights. Daily oral administration of WZYZP for 21 days started from the day after irradiation. On day 21, blood samples were collected by orbital venous phlebotom for hormone determination. Then the mice were killed by cervical dislocation. The testicular, epididymal, prostatic and seminal vesicle tissues were collected and weighted. Then the epididymides were used for sperm count, motility and abnormal rate examination immediately. Meanwhile, one part of testes was homogenized for biochemical analysis and others were fixed in 10% formalin for histological analysis.

### Biochemical analysis

The testicular tissue was homogenized with ice-cold saline to be 10% (w/v) homogenates. Protein concentration was determined by the Coomassie blue protein-binding using bovine serum albumin as a standard. Malondialdehyde (MDA) in homogenized testis tissue was determined using an assay kit (Beyotime Biotechnology, China). Total oxidant status (TOS) and Total Antioxidant Status (TAS) of the tissues were measured by the colorimetric methods described by Erel [12]. TAS results are expressed as μmol Trolox Equivalence per gram of tissue and TOS results are expressed as μmol H_2_O_2_ Equivalence per gram of tissue. Oxidative stress index (OSI) was calculated according to the following formula: OSI (Arbitrary Unit) = TOS/TAS [13].

### Sperm count, motility and abnormal rate assays

The epididymal sperm count and abnormal sperm rate was determined by a modified method [14]. Briefly, a 5 μl sperm sample was diluted with 95 μl diluent (5 g sodium bicarbonate, 1 ml 35% formalin and 25 mg eosin per 100 ml of distilled water). 10 μl of the thoroughly mixed diluted sperm suspension was transferred to each counting chamber of the neubauer hemocytometer. For determining the abnormal sperm rate, the fixed sperm suspension was placed on the slide and covered with a cover-slip. Approximately 200 sperm cells of each sample were examined under an optical microscope. The sperm were classified as normal, head abnormal or tail abnormal.

The progressive sperm motility was evaluated by a modified method [15]. In brief, a 5 μl sperm sample was diluted with 95 μl Hank’s balanced salt solution. An aliquot of the sperm suspension was placed on the slide and covered with a cover-slip. For each sample, approximately 200 sperm cells were examined under an optical microscope. The sperm were classified as motile or immotile.

### Serum hormone level assay

Blood samples were collected by orbital venous phlebotomy, and then centrifuged at 1000 × g at 4 °C for 10 min for serum separation. The levels of serum testosterone (T), luteinizing hormone (LH) and follicule-stimulating hormone (FSH) were determined using traditional radioimmunoassay methods and were performed according to the manufacturer’s instructions (Diagnostic System Laboratories, Webster, TX, USA).

### Morphology

Formalin-fixed tissue samples were dehydrated through an upgraded ethanol series, embedded in paraffin blocks and sectioned at 3 mm. Ultrathin sections of 5 μm were dewaxed by xylene, hydrated through a degraded ethanol series, and stained with hematoxylin-eosin. Then they were examined by a pathologist blinded to the treatments under an optical microscopy (Olympus, BX-51).

### Western blot analysis

The testes were dissected out and homogenized in pre-chilled RIPA buffer (50 mM Tris–HCl, 150 mM NaCl, 0.5% Sodium deoxicholate, 0.1% SDS, 1% Tween-100, 5 mM EDTA, 1 mM EGTA, 1 mM PMSF). The lysate were centrifuged at 12,000 g for 15 min at 4 °C to pellet the debris. Supernatant solutions were collected as whole cytoplasm protein for analyzing PCNA. Protein concentrations were determined by the Coomassie blue protein binding method using bovine serum albumin as standard. Then samples containing equal amounts of protein (50 μg) were boiled in protein loading buffer for 10 min, separated on 12% SDS-polyacrylamide gels and transferred to PVDF membranes. The membranes were blocked with 3% BSA in Tris-buffer saline. Then the membranes were incubated at 4 °C overnight with the PCNA antibody (1:1000) and β-actin antibody (1:1000), respectively (Santa Cruz Biotechnologies, Santa Cruz, CA). After washing, the membranes were incubated with a horseradish peroxidase conjugated secondary antibody (mouse anti-rabbit IgG, 1:5000; rabbit anti-goat IgG, 1:5000) (Santa Cruz Biotechnologies, Santa Cruz, CA) for 1 h at room temperature and were visualized using an enzyme-linked chemiluminescence reaction by imager instrument (Fujifilm, Las-3000). Relative intensities of the bands were quantified by Image-Pro Express 6.0 software.

### Statistical analysis

All the data were expressed as means ± standard deviation (SD). Differences between groups were assessed by one-way ANOVA and Tukey’s HSD test. A value of *p < 0.05* was considered statistically significant.

## Results

### Effects on testicular and accessory sexual organ weight

The body weight among four groups at beginning and the end was similar with no significance (data not shown). As shown in Table 2, the testis weight in model group was drastically reduced compared with that in control group (*p* < 0.05). WZYZP treatment restrained the decrease in testis induced by irradiation and there was significant difference between high dose group and model group (*p* < 0.05). Additionally, pelvic exposure to x-ray irradiation had no obvious effects on the seminal vesicle-prostate or epididymis weight.

**Table 2:** Effects of WZYZP on testicular and accessory sexual organ weight

Group	Dose (g/kg)	Testes	Epididymis	Seminal vesicle-prostate
Sham	-	0.54 ± 0.16	0.56 ± 0.06	0.68 ± 0.14
Model	-	0.35 ± 0.06^a^	0.54 ± 0.12	0.67 ± 0.07
WZYZP-L	0.25	0.39 ± 0.05	0.58 ± 0.16	0.65 ± 0.08
WZYZP-H	1.0	0.45 ± 0.03^b^	0.57 ± 0.09	0.71 ± 0.16

^*a*^ *p < 0.01* compared with the sham group; ^*b*^ *p < 0.05* compared with the model group

### Effects on sperm count, motility and abnormality

As shown in Table 3, sperm count and motility were significantly reduced, while sperm abnormality was increased in the irradiation group compared with those in control group (*p* < 0.01, *p* < 0.05, *p* < 0.05). After treatment with WZYZP for 3 weeks, sperm count in WZYZP-treated groups were significantly increased compared to model group (*p* < 0.05, *p* < 0.01). Meanwhile, sperm motility in the WZYZP-treated group was significantly higher than in the irradiation control (*p* < 0.05). Additionally, WZYZP treatment had no significant effect on the frequency of abnormal sperm.

**Table 3:** Effects of WZYZP on sperm count, motility and abnormality

Group	Count (10^6^/ml)	Motility (%)	Abnormality (%)
Sham	251.3 ± 32.6	92.1 ± 2.8	1.8 ± 0.8
Model	161.1 ± 21.1^b^	63.5 ± 6.8^a^	6.4 ± 0.8^a^
WZYZP-L	192.7 ± 28.6^c^	69.6 ± 7.9	6.5 ± 2.7
WZYZP-H	236.9 ± 15.7^d^	77.6 ± 2.1^c^	5.7 ± 1.7

^*a*^ *P < 0.05,* ^*b*^ *P < 0.01* compared with the sham group; ^*c*^ *P < 0.05,* ^*d*^ *P < 0.01* compared with the model group

### Effects on MDA, TOS and TAS in testes tissue

The oxidant-antioxidant status of the testes subjected to x-ray irradiation was assessed by determining the level of malondialdehyde (MDA), Total oxidant status (TOS) and Total Antioxidant Status (TAS). As shown in Table 4, MDA and OSI in model group was significantly increase higher than that in control group (*p* < 0.05, *p* < 0.01). After treatment with 2 g/kg WZYZP for 3 weeks, both of MDA level and OSI were significantly decreased compared to model group (*p* < 0.05, *p* < 0.01).

**Table 4:** Effects of WZYZP on testes oxidant and antioxidant status

Group	MDA (μM)	TAS (mmol Trolox Equiv./L)	TOS (mmol H_2_O_2_ Equiv./L)	OSI
Sham	1.24 ± 0.18	4.21 ± 0.28	114.8 ± 19.7	27.4 ± 6.2
Model	2.11 ± 0.24^a^	2.16 ± 0.24^a^	179.6 ± 21.1^a^	83.3 ± 18.9^b^
WZYZP-L	1.95 ± 0.21	3.18 ± 0.21	161.2 ± 14.4^c^	50.9 ± 10.3^c^
WZYZP-H	1.76 ± 0.05^c^	3.72 ± 0.85^c^	145.4 ± 18.5^c^	39.5 ± 14.8^d^

^*a*^ *P < 0.05,* ^*b*^ *P < 0.01* compared with the sham group; ^*c*^ *P < 0.05,* ^*d*^ *P < 0.01* compared with the model group

### Effects on serum hormones levels

As shown in Table 5, serum testosterone (T) in x-ray irradiation group was significantly lower than that in control group (*P < 0.01*). However, there were no significant differences in terms of luteinizing hormone (LH) and follicule-stimulating hormone (FSH). After treatment with WZYZP for 21 days, the serum testosterone level was higher compared to model group (*P < 0.05*). Meanwhile, WZYZP treatment had no effect on serum luteinizing hormone (LH) and follicule-stimulating hormone (FSH).

**Table 5:** Effects of WZYZP on testosterone (T), luteinizing hormone (LH) and follicule-stimulating hormone (FSH)

Group	T (nmol/L)	LH (m U/ml)	FSH (m U/ml)
Sham	16.41 ± 5.93	14.1 ± 3.2	5.49 ± 0.71
Model	6.71 ± 1.34^b^	13.3 ± 5.6	6.27 ± 1.24
WZYZP-L	9.47 ± 3.42	13.9 ± 4.1	5.63 ± 0.44
WZYZP-H	13.73 ± 4.15^c^	14.5 ± 5.7	6.41 ± 0.85

^*b*^ *P < 0.01* compared with the sham group;^*c*^ *P < 0.05* compared with the model group

### Effects on alterations of testicular architecture

**Fig. 1:**
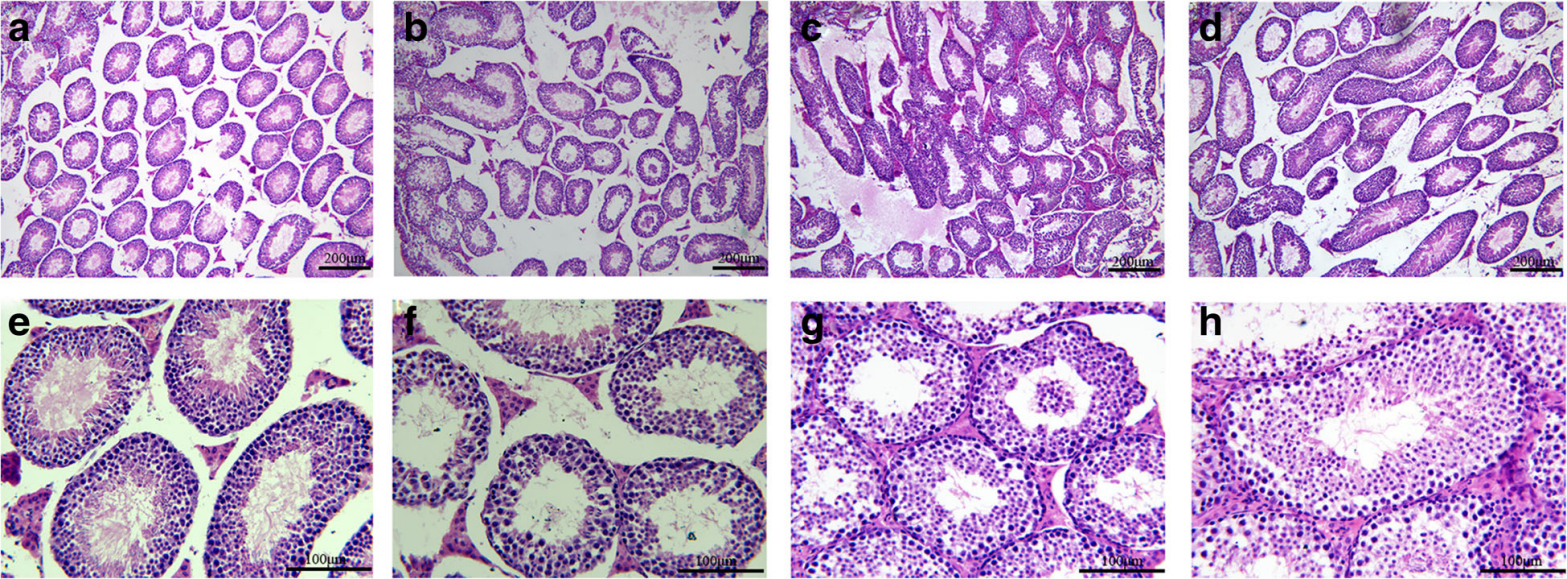
Modulation of radiation-induced histological changes in testes of mice by WZYZP. Representative photomicrographs of testis tissue from control (**a**, **e**), irradiation (**b**, **f**), irradiation + 0.25 g/kg (**c**, **g**) and 1 g/kg WZYZP treatment (**d**, **h**) groups. Above, magnification × 100; below, magnification × 400

As shown in Fig. 1, Testicular tissues in the control showed a normal arrangement of germinal and sertoli cells without any histopathological lesions. Distorted architecture of seminiferous tubules was seen in the form of shrunken tubules, exfoliation, intertubular oedema karyorrhexis, karyolysis, pycnotic nuclei, necrotic cells, and with degranulated cytoplasm in irradiated mice. WZYZP treatment rendered the quality as evident in the form of intact germinal epithelium, mild cytoplasmic vacuolization with the absence of karyolysis, pyknosis, and necrosis as well as increased germ cells number, and almost a normal testicular architecture was visualized by the end of experiment.

### Effects on expression of proliferating cell nuclear antigen (PCNA) in testes

**Fig. 2:**
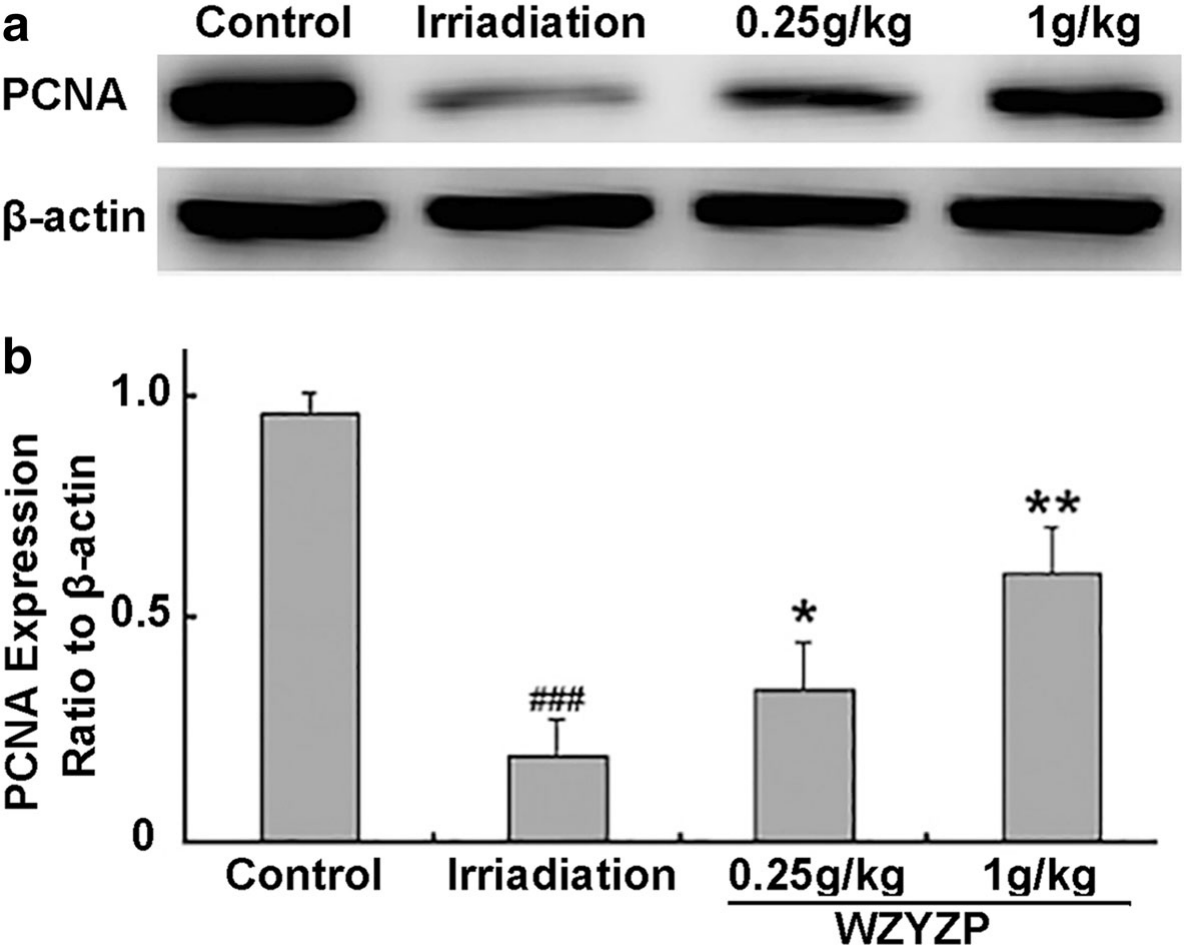
Effect of WZYZP treatment on PCNA protein expression of testes in mice exposed to x-ray irradiation. The relative optical density was normalized to β-actin. (**a**) Representative western blot of PCNA. (**b**) Quantification of the intensities of PCNA. (^###^ *P* < 0.001 compared with Control; *P < 0.05, **P < 0.01 compared with irradiation)

To explore the mechanisms underlying the protective effects of WZYZP against testicular damage in mice induced by ionizing radiation, we examined the expressions of PCNA in testes. PCNA, a nuclear protein and a co-factor for DNA polymerase δ, is reported to be involved in the RAD6-dependent DNA repair pathway in response to oxidative DNA damage [16]. As shown in Fig. 2a, the levels of PCNA were significantly decreased in irradiated group compared to control group (*p* < 0.001). However, WZYZP could effectively prevent x-ray induced downregulation of PCNA, which was important for both the DNA repair and spermatogenic cell proliferation.

## Discussion

The testis comprises two distinct compartments, the seminiferous tubules (the spermatogenesis site) and the Leydig cells (the testosterone source). Spermatogenesis is a highly complex process regulated by testicular cells such as various stage germ cells, Sertoli cells, Leydig cells and peritubular cells [17]. The testis is one of the most radiosensitive organs, which can be significantly functionally impaired by even very low doses of radiation [18]. In this study, testicular atrophy associated with distorted architecture of seminiferous tubules was observed in ionizing radiation mice at 21 days post-radiation. Meanwhile, the sperm count and motility were significantly decreased, and abnormal sperm rate was significantly increased. These results are consistent with previous reports [19, 20]. Administrated of WZYZP restrained the decrease in testis weight, improved the distorted architecture of seminiferous tubules, and reversed the decline of sperm quantity and quality but had no effect on the frequency of abnormal sperm.

It is well known that testosterone mainly secreted from the interstitial Leydig cells plays a key role in the development of male reproductive tissues such as the testis and prostate [21]. A reduction in testosterone levels can lead to the disruption of spermatogenesis. Also, its reduction implies injury to the testicular function [22]. It was demonstrated that pelvic exposure to x-ray irradiation could significantly lower the serum testosterone levels in male mice, leading to spermatogenic disorders. Treatment with WZYZP by gastric gavage restored testosterone levels to those of normal controls.

As a physical factor common in the natural world, ionizing irradiation could induce the body to generate large amounts of oxygen free radicals, causing severe lipid peroxidation, damage to macromolecules such as nucleic acids, proteins and enzymes, and ultimately cause damage to cells and tissues [23]. Testis is a highly prolific tissue with fast cellular renewal system along with rich in polyunsaturated fatty acids and poor antioxidant defense, and for these reasons it becomes an easy target for the radiation-induced free radicals mediated damage. Excess amounts of reactive oxygen radicals induce production of abnormal sperms and infertility [24]. In this study, oxidative stress was observed in irradiated testes tissue in terms of evaluated MDA and OSI. After treatment with 1 g/kg WZYZP for 3 weeks, both of MDA level and OSI were significantly decreased compared to the irradiated mice. We hypothesized that WZYZP removes free radicals by creating an antioxidative environment within cells, alleviating the damage induced by free radicals, and thus reducing damage to testicular cell DNA. This process rescues the defects in testicular spermatogenesis and sperm quantity and quality caused by irradiation.

Progression of cell cycle is important for proliferating spermatogenic cells. PCNA, a nuclear antigen in proliferating cells, is involved in DNA replication in proliferating spermatogonia [25]. Subsequently, this protein described as a cofactor for DNA polymerase δ, which is involved in the control of DNA replication and repair. PCNA protein levels rise only during the S-phase of the cell cycle, and form complex with p21 inhibitor. PCNA protein ubiquitinated and involved in the RAD6-dependent DNA repair pathway in response to oxidative DNA damage. In the present study, WZYZP treatment enhanced the expression of PCNA protein in response to irradiation-induced DNA damage.

## Conclusions

Our results demonstrated that Chinese medicine WZYZP improve testes weight and sperm quality, as well as seminiferous tubule structure in this study, which indicated that its radioprotective effect against testicular damage induced by x-rays irradiation in mice. Meanwhile, WZYZP significantly decreased the oxidative stress and enhanced the PCNA expression in testes, which might be involved in the radioprotective effect. However, the precise mechanism still need to be further investigated in the future.

## Abbreviations

FSH: follicule-stimulating hormone
LH: luteinizing hormone
MDA: Malondialdehyde
OSI: Oxidative stress index
PCNA: Proliferating cell nuclear antigen
T: Testosterone
TAS: Total Antioxidant Status
TOS: Total oxidant status
WZYZP: Wuzi Yzong pill
